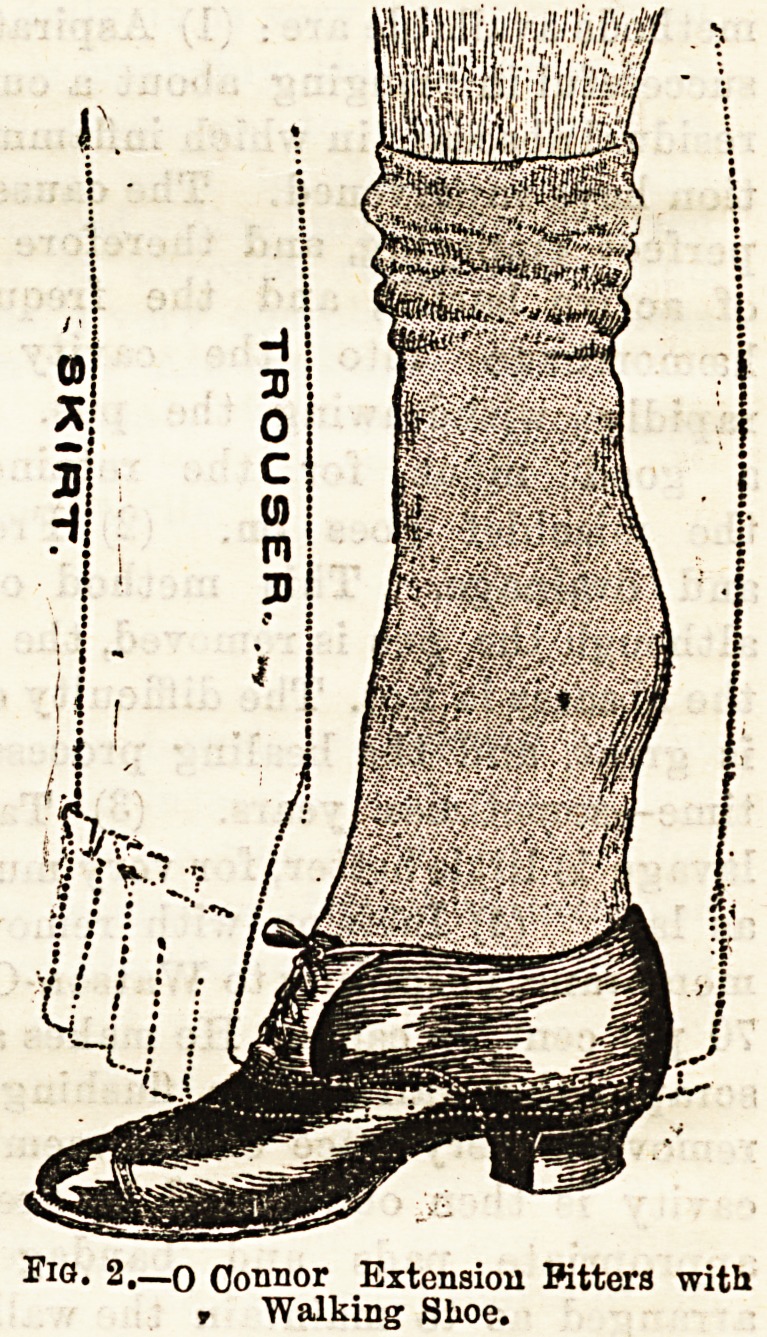# New Appliances and Things Medical

**Published:** 1896-01-18

**Authors:** 


### NEW APPLIANCES AND THINGS MEDICAL.

### THE O'CONNOR EXTENSION AND CLYDE SUPPORT.

(THE O'CONNOR EXTENSION COMPANY, 2, BLOOMSBURY STREET, W.C.)

The O'Connor Extension is a new contrivance for obviating the discomforts and unsightliness attendant on shortening of the lower extremity. The principles on which the methods are founded are physiologically sound, and, what is more important, the contrivance itself has practical applications which have already met with sufficient success to justify confidence in its ultimate wide adoption in cases for which it is intended. The contrivance may be described as a false boot in which the heel of the offending foot occupies the position usually taken by the ankle, and the toes the normal position of the instep (Fig. 1). As may be seen from the diagram, the foot is fully extended on the ankle, the heel being supported by appropriate building up of the interior of the boot. The necessity for "toe-walking" and its attendant evils is thus obviated, and the unpleasing appearance of cork sole, iron braces, or stirrups rendered unnecessary. To secure perfect similarity between the two feet a boot or shoe, as worn on the sound foot, may be slipped over the "Extension" (Fig. 2). Its application is for all cases of compensatory talipes equinus, or for those cases in which, from whatever causes, there is a real or apparent shortening of over three inches. We have been assured by those who wear the "Extension" that as far as comfort is concerned little is left to be desired, and from personal observation we can testify that there is nothing to offend the eye or attract attention in the general appearance. Those who wear this boot for the first time must be prepared for a certain degree of uneasiness, owing to the extended position of the foot and the relaxation of the Tendo Achilles. The discomfort, however, passes off in a few days, and the tendon accommodates itself to the new position, and becomes shorter by a process of physiological adaptiveness. In his book on deformities of the foot, Mr. Walsham speaks highly of the method for compensatory equinus, and we believe that, by a discriminating selection of cases, and the exercise of a little patience on the part of the wearers, the contrivance has a great future before it.

FIG. 1.—Dotted outlines to show position occupied by foot.

FIG. 3.—The Clyde Ankle Support.

FIG. 2.—O Connor Extension Fitters with Walking Shoe.

### THE CLYDE ANKLE SUPPORT.

The Clyde ankle support is another ingenious invention introduced by the same firm, and intended to replace the cumbrous and ungainly irons which heretofore have been almost the sole resource of surgeons in their treatment of weak ankles, whether from constitutional causes, or from the various forms of paralysis or talipes which are accompanied by this intractable condition. The contrivance is simplicity itself. A careful mould is taken of the ankle and foot in a plastic material, which is then made rigid and allowed to constitute the basis on which the leather of the "uppers" is sewn. The method ensures absolute accuracy of fit, and from the nature of the material the support to the ankle is as complete as a plaster of Paris splint (Fig. 3). The boot itself presents no dissimilarity from an ordinary boot, and is perfectly comfortable to wear, and as time goes on adapts itself even more completely to the minor irregularities in contour which may have been neglected in the original moulding. It is obvious that in skilful hands this ankle support has many practical applications in combination with a properly-shaped false sole or instep arch to meet the requirements of those cases of weak ankles which are complicated with other deformities or malformations of the foot.

## Figures and Tables

**Fig. 1. f1:**
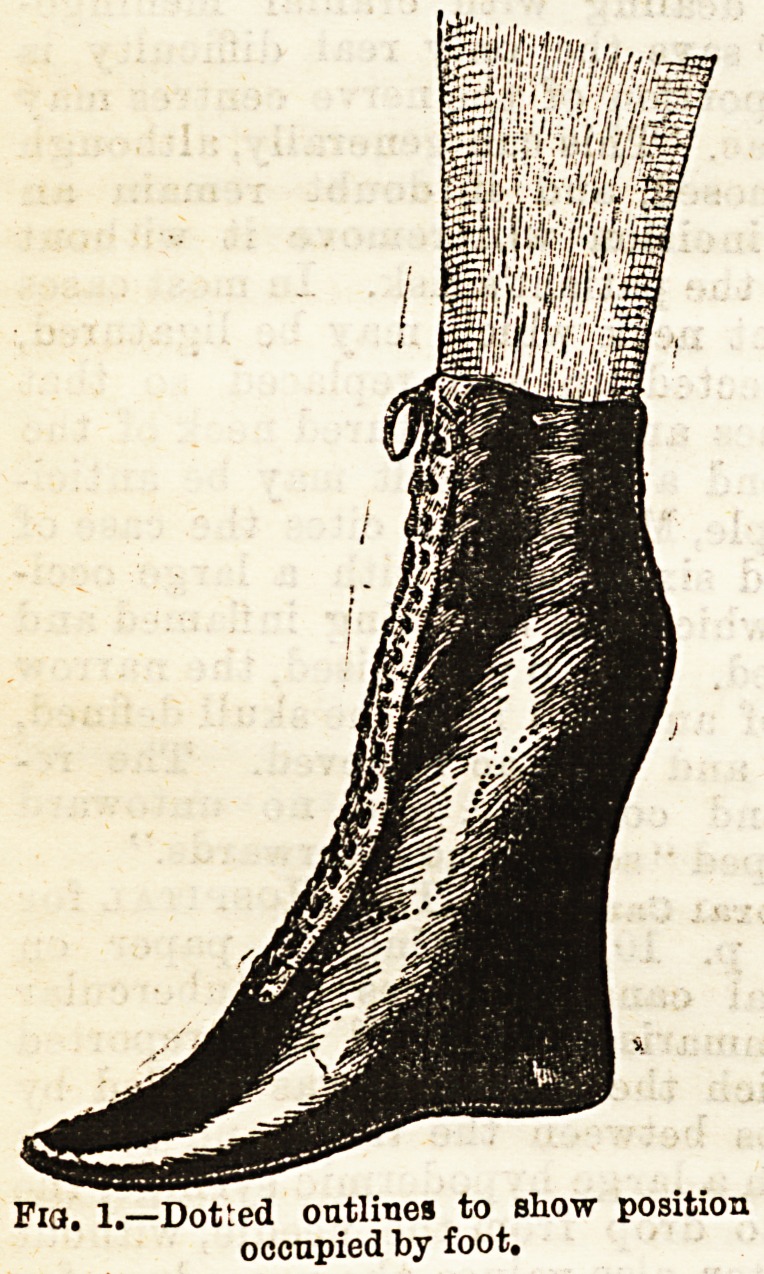


**Fig. 3. f2:**
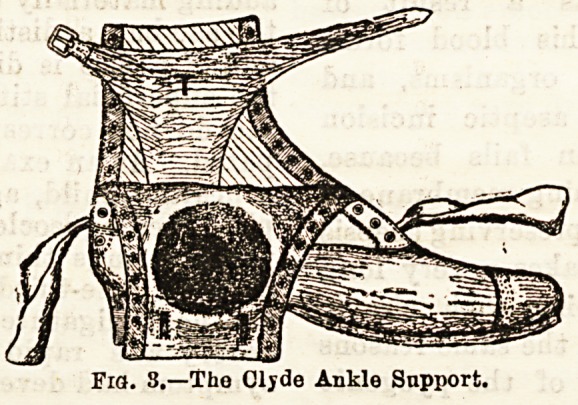


**Fig. 2. f3:**